# A High-Resolution Imaging Method for Multiple-Input Multiple-Output Sonar Based on Deterministic Compressed Sensing

**DOI:** 10.3390/s24041296

**Published:** 2024-02-17

**Authors:** Ning Gao, Feng Xu, Juan Yang

**Affiliations:** 1 Ocean Acoustic Technology Laboratory, Institute of Acoustics, Chinese Academy of Sciences, Beijing 100190, China; gaoning@mail.ioa.ac.cn (N.G.); xf@mail.ioa.ac.cn (F.X.); 2 School of Electronic, Electrical and Communication Engineering, University of Chinese Academy of Sciences, Beijing 100049, China

**Keywords:** MIMO sonar, high resolution, matched filtering, compressed sensing, signal reconstruction

## Abstract

Differences between conventional sonar and Multiple-Input Multiple-Output (MIMO) sonar systems arise in achieving high angular and range resolution. MIMO sonar uses Matched Filtering (MF) with well-correlated transmitted signals to enhance spatial resolution by obtaining virtual arrays. However, imperfect correlation characteristics yield high sidelobe values, which hinder accurate target localization in underwater imagery. To address this, a Compressed Sensing (CS) method is proposed by reconstructing echo signals to suppress correlation noise between orthogonal waveforms. A shifted dictionary matrix and a deterministic Discrete Fourier Transform (DFT) measurement matrix are used to multiply received echo signals to yield compressed measurements. A sparse recovery algorithm is applied to optimize signal reconstruction before joint transmit–receive beamforming forms a 2D sonar image in the angle-range domain. Numerical simulations and lake experimental results confirm the effectiveness of the proposed method, by obtaining a lower sidelobe sonar image under sub-Nyquist sampling rates as compared with other approaches.

## 1. Introduction

The concept of Multiple-Input Multiple-Output (MIMO) systems originated in the field of communications. Expanding its applicability to sonar detection systems, the MIMO concept in conjunction with the idea of diversity gain, has facilitated the development of various MIMO detection systems, including single-site, dual-site, and hybrid multi-site configurations. The application of MIMO technology has yielded significant advancements in radar and sonar array signal processing, particularly in the domain of small target detection [1,2,3]. The core principles of MIMO technology lie in waveform diversity and spatial diversity [4,5], which are used in dense MIMO sonar and distributed MIMO sonar systems, respectively. Distributed MIMO systems offer spatial diversity for achieving stable target detection and tracking. A notable application of distributed MIMO sonar systems in enhancing target detection capabilities is demonstrated by Vossen et al. in their work on estimating underwater geological features [6]. Dense MIMO sonar systems, on the other hand, use simultaneous orthogonal waveform transmission at the transmitter and use Matched Filtering (MF) techniques at the receiver to process echo signals. Waveform diversity enhances the virtual aperture of the receiving array and improves the performance of underwater imaging [7,8,9]. The equivalence between MIMO sonar and virtual Single-Input Multiple-Output (SIMO) sonar counterparts can be satisfied only when transmitted signals demonstrate favorable correlation properties [10]. The integration of MIMO technology into the domain of sonar imaging offers potential advantages, such as reducing the number of required array elements and simplifying system hardware.

In the context of two-dimensional MIMO imaging sonar, a virtual array with a larger aperture is formed by placing two transmitters at opposite ends of multiple receivers. This arrangement is intended to improve the spatial resolution of sonar images [11,12]. To ensure coherent summation of echoes within the receiver, it is essential that independently transmitted orthogonal waveforms utilize identical bandwidth resources. Hao He et al. conducted waveform design for the transmission beamforming of wideband signals in MIMO sonar systems [13]. In addition, the presence of Fano- and electromagnetically induced transparency (EIT)-type transmission line shapes have been observed to produce high-quality (Q) factors, which are useful for high-sensitivity sensing in the development of sensors [14]. Currently, Fano resonances and EIT have demonstrated good resonance response in metamaterial units and square lattice plasma nanostructures [15,16]. Compared with the CS method, its low computational complexity reduces the cost of practical application.

Disregarding the Doppler frequency shift in the echoes, the MF procedure at the receiver can leverage correlation processing between the transmitted signals and echoes [9]. The calculated autocorrelation functions (ACFs) and cross-correlation functions (CCFs) are added by incorporating delay and attenuation adjustments for various targets. Achieving a high main lobe to sidelobe ratio in the range dimension becomes challenging because of high-level inter-waveform cross-correlation. For Linear Frequency Modulation (LFM, or Chirp) waveforms, the Time-Bandwidth Product (TBP) of the frequency sweep can be adjusted to modify the ratio between CCF and ACF levels, effectively reducing the impact of sidelobes. As the length of the encoded waveforms increases, the level of sidelobes decreases. However, a concomitant increase in the length of the receivers leads to a substantial surge in computational requirements, rendering it difficult to apply in the field of sonar imaging [17]. Li Jian et al. presented the design of an optimal receiver filter with significantly reduced range sidelobes [8]. But the complex iterative computation poses certain limitations on the inherent reduction in sidelobes in imaging results. Multiple orthogonal waveforms and separation methods have been proposed in the field of imaging for MIMO-SAR [18], but their practical application in sonar systems is challenging. Although considerable progress has been made in improving the detection performance of MIMO sonar systems [19,20], research on suppressing sidelobe levels in improving sonar imaging remains limited.

Compressive Sensing (CS) offers the capability to subsample sparse signals [21], enabling compression of signals during observation at sampling rates much lower than the Nyquist rate. By collecting samples from the compressed signal, the target range can be directly estimated from the sparse domain, allowing for target detection in the received echo signals with reduced measurement requirements [22]. In this study, an effective method based on CS to mitigate sidelobes interference in MIMO sonar imaging is proposed. In noise-free conditions, the output of CS exhibits no autocorrelation sidelobes. In the presence of noise, it is hypothesized that CS can also mitigate the inter-waveform cross-correlation levels. This enables the detection of two closely spaced targets with higher resolution compared with conventional MF [23,24].

The rest of this paper is organized as follows. Section 2 introduces the method adopted in our study. Section 3 describes the numerical simulation and experimental results, which are analyzed in subsections. Finally, in Section 4, we conclude this article with a summary.

## 2. Methods

### 2.1. MIMO Sonar Array Layout

Considering the coordinate origin as the center, a MIMO sonar array comprises a transmitting uniform linear array (ULA) with Mt elements and a receiving ULA with Nr elements. The inter-element spacing between the transmitting sensors and receiving sensors is denoted as *d_t_* and *d_r_*, respectively, both equivalent to half-wavelength intervals. By approximating the array equivalent phase center, the virtual array is constructed to expand the receiving array. The specific combination of the transmitting and receiving arrays gives rise to different MIMO array configurations, each characterized by a distinct virtual array representation. To maximize the effective aperture of the MIMO system, it is imperative to ensure that the virtual elements remain suitably separated [9]. The spacing constraints between *d_t_* and *d_r_* in the MIMO array normally satisfy $d_{t}=N_{r}d_{r}$.

The MIMO array is strategically arranged to achieve optimal azimuthal resolution while minimizing the physical size. Assuming an array configuration and sensor count, the physical size of the MIMO array is denoted as $\left(M_{t}-1\right)N_{r}d_{r}$. The effective aperture DM of the MIMO array is equivalent to the aperture of the virtual SIMO sonar, given by $\left(M_{t}N_{r}-1\right)d_{r}$. The SIMO array effective aperture *D_S_* is identical to its physical size. When the physical dimensions of the MIMO and SIMO arrays are equal, the relationship between their effective apertures can be expressed as

(1) $$D_{M\;}=\frac{M_{t}N_{r}-1}{\left(M_{t}-1\right)N_{r}}D_{S\;}$$

(2) $$\frac{D_{M\;}}{D_{S\;}}=\frac{M_{t}N_{r}-1}{\left(M_{t}-1\right)N_{r}}=\frac{M_{t}-\frac{1}{N_{r}}}{\left(M_{t}-1\right)}\approx \frac{M_{t}}{M_{t}-1}$$

To ensure a satisfactory level of angular resolution, it is common practice to assign a sufficiently large number of receiving sensors *N_r_* to the MIMO array. The ratio of effective apertures is directly influenced by the number of transmitting elements *M_t_*. As the number of transmitting elements *M_t_* increases in the MIMO array, the effective aperture of the array gradually converges towards its own physical size. A reduction in the efficiency of aperture expansion at the receiving end is inevitable. Remarkably, when the number of transmitting elements *M_t_* is set to 2, the MIMO array achieves its optimal configuration with the maximum effective aperture, surpassing that of other MIMO arrays.

**Figure 1:**
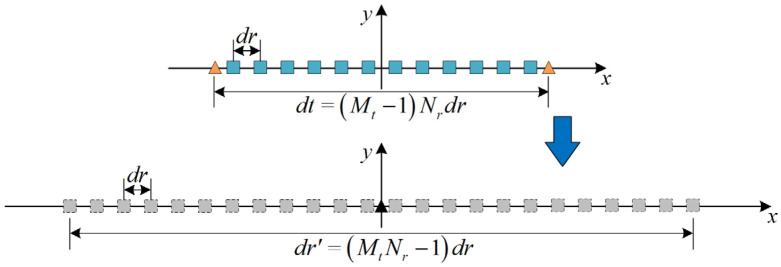
Equivalent procedure of the MIMO virtual array.

Figure 1 shows an extended MIMO imaging sonar system with two transmitter subarrays positioned at the ends of the receiving ULA in engineering applications. The transmission of multiple orthogonal waveforms enables the derivation of analytical coordinates with 2*N_r_* virtual array elements, which is predicted by the virtual array expansion theory. This advantageous configuration facilitates improved angular resolution and enhances the imaging performance of the sonar system.

### 2.2. Signal Model

Suppose that two transmitting sensors simultaneously emit narrowband LFM pulses with the same pulse duration (*T*) and bandwidth (*B*). The frequency sweep rate denoted by K=B/T remains constant for both pulses but differs in the modulation direction. Considering the relatively low velocity between the MIMO sonar and the target, the influence of the Doppler frequency shift can be disregarded. To simplify the analysis, we assume the target to be an ideal scatter, neglecting any medium absorption and transmission losses associated with free-space propagation [25]. Under the assumption of far-field conditions, we consider a single target located at (*P*_0_, 0), where the echo signals reflected from the target and received by Nr receiving sensors can be assumed to be fully correlated. Mathematically, this can be expressed as the baseband sum of the received echo signal at the *n*th receiver:

(3) $$x_{n}\left(t\right)=\overset{M_{t}}{\underset{m=1}{\sum }}\sigma_{m}^{P_{0}}s_{m}\left(t-\tau_{tm}-\tau_{rn}\right)+\eta_{0}\left(t\right)$$

where $\sigma_{m}^{P_{0}}$ denotes the scattering coefficient of the target for the *m*th waveform, $\tau_{tm}$ represents the time delay of sound propagation from the *m*th transmitting sensor to the target scatter, $\tau_{rn}$ denotes the time delay from the target to the *n*th receiving sensor, and $\eta_{0}\left(t\right)$ represents the Gaussian white noise at the nth receiving sensor, which is uncorrelated with the two transmitting LFM pulses.

Traditional methods in MIMO imaging sonar use multiple sets of matched filters for the purpose of separating superposed echo signals [15]. These filters are utilized to generate MIMO sonar images by applying digital beamforming to both the transmitter and receiver, contributing to the acquisition of multiple beams. Each filter’s impulse response function corresponds to a specific transmitted waveform, which can be expressed as

(4) $$\mu_{m}\left(t\right)={\left[s_{m}\left(T_{0}-t\right)\right]}^{*}$$

where ${\left[\cdot \right]}^{*}$ denotes conjugation. Specifically, by applying the *m*th matched filter to the echo signal received at the *n*th sensing element, the resulting filtered signal can be expressed as

(5) $$\begin{array}{ll} y_{mn}\left(t\right) & =x_{n}\left(t\right)*\mu_{m}\left(t\right)\\ & =\overset{M_{t}}{\underset{m=1}{\sum }}\sigma_{m}^{P_{0}}s_{m}\left(t-\tau_{tm}-\tau_{rn}\right)*\mu_{m}\left(t\right)+\eta_{0}\left(t\right)*\mu_{m}\left(t\right)\\ & =\overset{M_{t}}{\underset{m=1}{\sum }}R_{m,m}\left(t-\tau_{tm}-\tau_{rn}-T_{0}\right)+\overset{M_{t}}{\underset{i=1,i\neq m}{\sum }}R_{m,i}\left(t-\tau_{ti}-\tau_{ri}-T_{0}\right) \end{array}$$

The noise component within the output of the filter is typically insignificant. The calculated autocorrelation functions (ACFs) of the *m*th transmitted waveform are denoted as $R_{m,m}$, while $R_{m,i}$ represents the cross-correlation functions (CCFs) between the *m*th and *i*th transmitted waveforms. This can be disregarded when its magnitude is sufficiently lower than the ACF. The quantitative analysis of signal ACFs and CCFs is discussed in [12]. The same frequency band and pulse width ensure their ACF exhibit identical characteristics. The expressions for the ACF and CCF are as follows:

(6) $$R_{m,m}\left(t\right)=\left(T_{0}-\left|t\right|\right)\mathrm{rect}\left(\frac{t}{2T_{0}}\right)\sin c\left[\pi K\left(T_{0}-\left|t\right|\right)t\right]\exp\left(j2\pi f_{0}t\right)$$

(7) $$R_{m,i}\left(t\right)\approx \frac{1}{T_{0}\sqrt{2K}}\mathrm{rect}\left(\frac{t}{2T_{0}}\right)\exp\left[j2\pi \left(f_{0}t+\frac{B}{2}t+\frac{1}{2}Kt^{2}\right)\right]$$

The cross-correlation noise of LFM waveforms is directly influenced by the Time-Bandwidth Product (TBP), while in the practical applications of sonar systems, limitations exist that prevent it from being infinitely large. LFM waveforms with a large TBP can mitigate the high sidelobe levels observed in the range dimension when using the MF method. The ratio between the maximum absolute values of the CCF and ACF is approximately

(8) $$\rho =\frac{max\left(\left|R_{m,m}\left(t\right)\right|\right)}{max\left(\left|R_{m,i}\left(t\right)\right|\right)}\approx \frac{1}{T_{0}\sqrt{2K}}=\frac{1}{\sqrt{2BT_{0}}}$$

**Figure 2:**
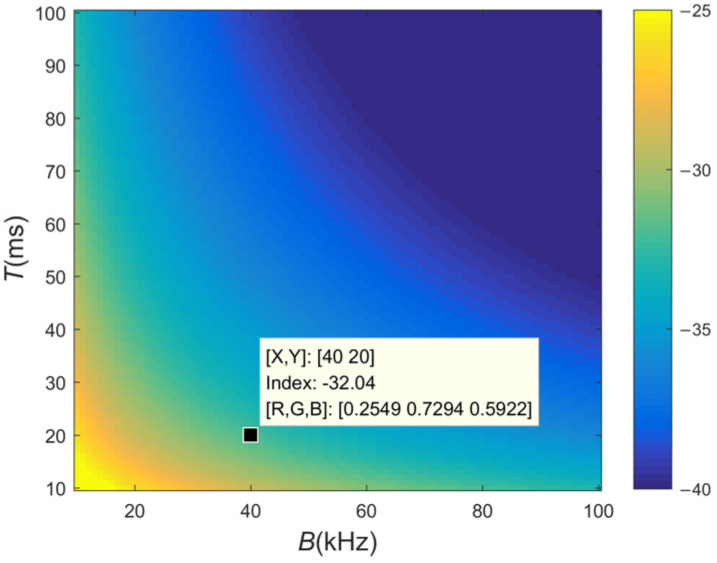
The ratio ρ at different pulse widths T and bandwidths B.

From Figure 2, it is evident that a larger TBP effectively reduces the levels of the CCF. For a given bandwidth, the ratio ρ decreases as the pulse width T increases, and vice versa. To provide a concrete example, consider a pair of LFM pulses with *B* = 40 kHz and *T* = 40 ms, where the CCF level is suppressed to approximately −35 dB. When *B* = 60 kHz, it is only when *T* ≥ 90 ms that the ratio drops to −40 dB. Nevertheless, shorter pulses are typically used for target detection in practical applications. Increasing both *B* and *T* would result in higher system costs and increased hardware complexity.

**Figure 3:**
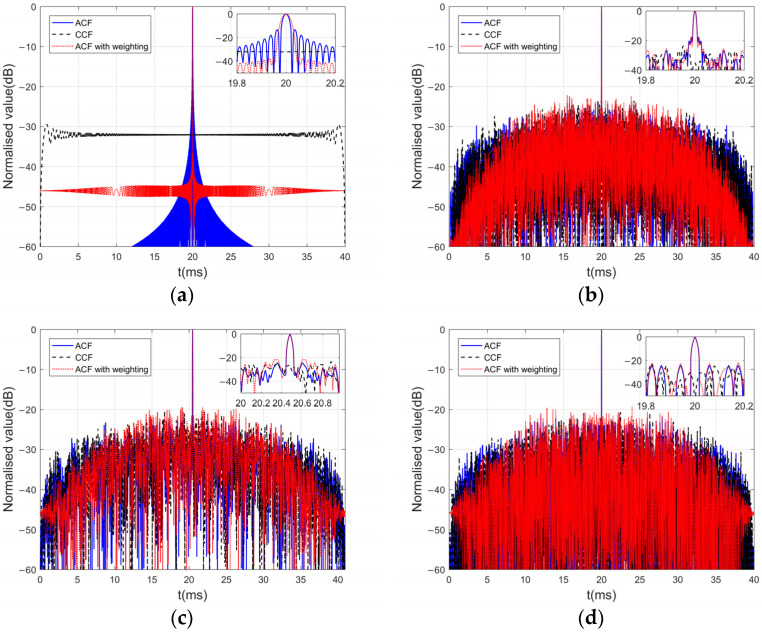
Comparison of the ACF and CCF in various transmitted signals: (**a**) chirp waveforms, (**b**) frequency-hopped chirp waveforms, (**c**) polyphase orthogonal code sequence waveforms, and (**d**) gold sequence waveforms.

In MIMO sonar systems, obtaining perfectly orthogonal waveforms is not feasible in practical applications. The degradation of image quality poses a challenge to the detection performance of sonar systems. Numerous scholars have used frequency-encoded or phase-encoded waveforms to achieve the design of orthogonal waveforms. Examples of such waveforms include frequency-hopped LFM waveforms [26], polyphase orthogonal code sequences signals [27], and gold sequences signals [28]. Figure 3 presents the results of the ACF and CCF by utilizing different transmission waveforms implemented with MF and window functions. In Figure 3a, up- and down-chirp waveforms are used, with a bandwidth of 40 kHz and a chirp duration of 20 ms. Figure 3b utilizes a CDMA waveform with LFM sub-pulses as described in [29], consisting of 100 subcodes and a total pulse duration of 20 ms. In Figure 3c, an orthogonal polyphase-coded waveform is utilized, featuring a sub-pulse width of 0.04 ms, 512 subcodes, and 8 random phase values. Lastly, Figure 3d displays a gold sequence signal constructed from a 10-stage m-sequence, with a total code length of 1024 and a time width of 20 ms.

Through comparison, it is evident that the sidelobe values of the ACF for the encoded signals (solid blue line) are significantly lower than those of the LFM waveforms. The correlation values between the up- and down-chirp waveforms exhibit considerable reduction, while the CCF between the encoded signals (dashed black line) is slightly higher in comparison. Additionally, the utilization of a Chebyshev window with LFM waveforms effectively reduces the sidelobe levels in the ACF (dashed red line). However, it does not improve (may even exacerbate) the sidelobe values of the ACF for the encoded signals. This limitation hampers their suitability as transmitted signals in MIMO sonar systems. The −3 dB main lobe widths of these waveforms are all below the millisecond level. While the encoded signals offer improved autocorrelation in practical applications, their correlation remains unimproved, and their hardware system complexity is higher in sonar systems. Hence, up- and down-LFM waveforms are generally chosen as the preferred transmitted waveform.

### 2.3. The Procedure of the Proposed Method

CS replaces the requirements of high-resolution sampling and data compression by combining the two steps into a single low-resolution acquisition step [30]. The successful recovery of the original signal using CS requires the fulfillment of two key conditions: sparsity and incoherence. A special measurement matrix that satisfies the restricted isometric property (RIP) [31,32] must be used. CS exploits the fact that a small set of nonadaptive linear measurements of a compressible signal carries enough information for reconstruction and processing [33]. The transmitted signal shifted dictionary matrix is simply generated to make the echo signal sparse representation. A computationally fast and efficient DFT deterministic matrix is constructed, and deterministic sub-sampling introduces randomness in a deterministic way so that the matrix does not need to be stored for reconstruction purposes. Due to the sampler’s deterministic concept, proposed deterministic construction may achieve some compression ratio and less computational complexity [30], leading to a reduction in samples than that required by random matrices. Also, orthonormalizing the measurement matrix makes it become mutually incoherent with any dictionary; thus, recovery is possible with high probability. CS technology allows for a significant reduction in the sampling rate far below the Nyquist rate [34]. It proves to be a promising approach for detecting Ultra-Wideband signals at reduced sampling rates, providing that the signals exhibit a sparse representation in a specific spatial domain. By exploiting only a few samples acquired from the echoes, CS fulfills effective target detection and estimation of the target distance from the transmitter.

**Figure 4:**
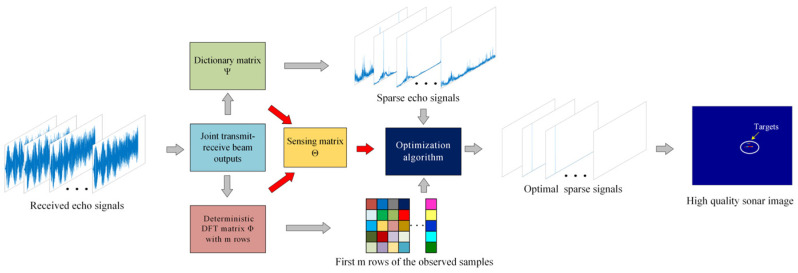
The processing procedure of the proposed method.

A flow chart of the proposed method is depicted in Figure 4, illustrating the joint transmit–receive beamforming implements increased angular resolution in the form of a multi-beam image. The dictionary matrix ensures the sparsity of the signal, while the measurement matrix guarantees the efficacy of signal reconstruction. These two matrices collectively form the sensing matrix as a pivotal constituent in the algorithm. By reconstructing the target echo signals, the algorithm effectively suppresses the inter-correlation noise among the received orthogonal waveforms, leading to a substantial reduction in sidelobe interference of sonar image. The use of an under-sampling rate is conducive to mitigate computational complexity, making it feasible for practical applications without significantly impacting storage requirements.

A joint transmit–receive beamforming is performed on the echo signals in order to form beams that encompass the target scattering points within the desired angular range. Supposing the *k*th beam output is represented by $B_{k}\left(t\right)\in {\mathbb{C}}^{L\times 1}$, where each beam output has a signal length of *L*, the beam outputs can be expressed in matrix form as:

(9) $$BF=\left[B_{1}{\left(t\right)}^{T},B_{2}{\left(t\right)}^{T},\cdot \cdot \cdot ,B_{k}{\left(t\right)}^{T}\right]\in {\mathbb{C}}^{K\times L}$$

A dictionary matrix is used for the purpose of sparse representation of the echo signals, capitalizing on the favorable intercorrelation existing between the up and down frequency signals. The echo signals predominantly manifest significant peaks at the positions corresponding to the autocorrelation while considering near-zero values at other positions. The sparsity level of the signal is mainly determined by the number of targets contained in the echo signals. Supposing the measurement sample is sufficient, the dictionary matrix ensures the number of distinguished targets. The dictionary matrix Ψ can be expressed in a specific manner as follows:

(10) $$\Psi =\left[\begin{array}{ccccc} S_{T}\left(t_{1}\right) & 0 & \cdots & 0 & 0\\ 0 & S_{T}\left(t_{2}\right) & 0 & \cdots & 0\\ \vdots & 0 & \ddots & \cdots & \vdots \\ 0 & \vdots & 0 & S_{T}\left(t_{l-1}\right) & 0\\ 0 & 0 & \cdots & 0 & S_{T}\left(t_{l}\right) \end{array}\right]\in {\mathbb{C}}^{\left(L-N_{T}\right)\times L}$$

where $S_{T}\left(t_{l}\right)$ denotes the transmit signal with element $\left(s_{1},s_{2}\cdots ,s_{n}\right)$, while $\left[S_{T}\left(t_{2}\right),\cdots ,S_{T}\left(t_{l}\right)\right]$ denotes the shifted version signals with elements $\left[\left(s_{n},s_{1},\cdots ,s_{n-1}\right),\cdots ,\left(s_{n},s_{n-1},\cdots ,s_{1}\right)\right]$. *L* and *N_T_* represent the number of samples in the echo signal and transmit signal, respectively. Assuming **X** and **Y** denote the echo signal and the sparse signal, the sparse representation process can be formulated as follows:

(11) Y=Ψ·X

Subsequently, the proposed method is utilized to acquire observation samples for each beam, with orthogonal normalization ensuring their efficacy. To alleviate computational complexity, the first M rows of the observation matrix $\Phi =e^{-\frac{i2\pi kj}{n}},k,j=0,1,\ldots n-1$ are selected. **Φ** can be mathematically expressed as:

(12) $$\Phi =\left[\Phi_{1},\Phi_{2},\cdot \cdot \cdot ,\Phi_{m}\right]\in {\mathbb{C}}^{M\times L}$$

where $\Phi_{m}\in {\mathbb{C}}^{1\times L}$; the kth beam is multiplied element-wise with the *m*th row of the measurement matrix to yield the observation samples for the kth direction:

(13) $$z_{k}\left(t\right)=\overset{M}{\underset{m=1}{\sum }}\Phi_{m}\cdot B_{k}\left(t\right)$$

The representation of the observation samples for the entire beam output can be expressed as:

(14) $$Z=\left[z_{0}{\left(t\right)}^{T},z_{1}{\left(t\right)}^{T},\cdot \cdot \cdot ,z_{k}{\left(t\right)}^{T}\right]\in {\mathbb{C}}^{K\times M}$$

The process of generating observation samples can be represented in matrix form as:

(15) $$Z=\Phi \cdot BF^{T}$$

According to the analysis and derivation in [35], the dictionary matrix **Ψ** and the measurement matrix **Φ** constitute the sensing matrix:

(16) $$\Theta =\Phi \cdot \Psi^{-1}$$

To obtain the optimal sparse solution, we seek the solution to the underdetermined linear equations by using Equation (17):

(17) Z=Θ·Y

**Figure 5:**
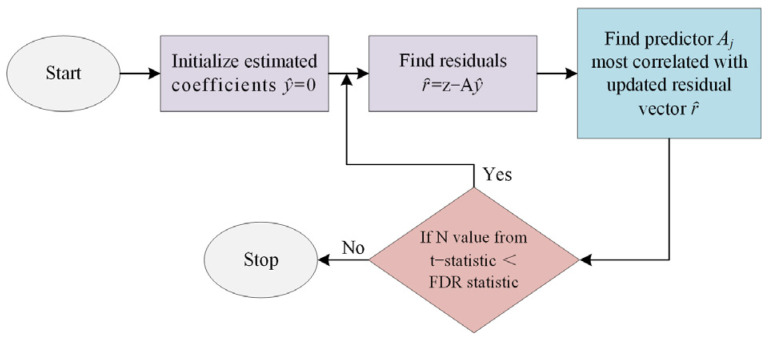
The procedure of the optimization algorithm.

Figure 5 shows the procedure of the optimization algorithm. To retrieve the target information, an optimization algorithm was used to optimize sparse signals, such as Basis Pursuit Denoising (BPDN) and Least Absolute Shrinkage and Selection Operator (LASSO) provided by the Sparse LAB package [24]. They are equivalent methods but were developed by different research communities.

(18) $$\underset{y}{min}∥y{∥}_{1}\;subject\;to\;∥Ay-z{∥}_{2}\leq \varepsilon$$

(19) $$\underset{y}{min}∥Ay-z{∥}_{2}\;subject\;to\;∥y{∥}_{1}\leq t$$

Equations (18) and (19) denote the objective functions BPDN and LASSO. respectively. Where $\;\;\varepsilon =m\sigma^{2}$, and $\sigma^{2}$ denotes the noise variance in Equation (18). Equation (19) was solved by the Stepwise regression False Discovery Rate (SWr-FDR) algorithm based on the absolute size of their t-statistic up to some preset significance threshold based on the False Discovery Rate (FDR).

## 3. Results and Discussion

This section mainly describes the performance of the proposed method compared with MF [12], MF with weighting, and the Richardson–Lucy (R-L) algorithm, also known as the Deconvolution (Dcv) algorithm [36], which are evaluated by analyzing simulation results and experimental data.

### 3.1. Numerical Simulation

To illustrate the effectiveness of the proposed method, we compare the peak sidelobe level (PSL) and the −3 dB main lobe width of the different methods. In addition, we use the normalized root mean square error (RMSE) as the metric to analyze the influence of observed samples’ quantity on the signal reconstruction process of the algorithm. Considering the target’s coefficient is 1, both up- and down-chirp waveforms were simultaneously emitted from two distinct transmitting elements. The numerical simulation parameters are shown in Table 1.

PSL is used as an evaluation index to assess the suppression of cross-correlation noise by output superposition signals, as shown by Equation (20)

(20) $$\mathrm{PSL}=\underset{\mathrm{SL}}{max}\tilde{z}\left(t\right)$$

where $\tilde{z}\left(t\right)$ represents the output signals, SL represents the level value of the sidelobe area, and $max\left(\cdot \right)$ represents the maximum value.

The evaluation metric used to compare the relative reconstruction errors of various measurement matrices under noisy conditions was the *RMSE*, as calculated according to the Formula (21).

(21) $$RMSE=\frac{∥z\left(t\right)-\tilde{z}\left(t\right){∥}_{2}}{∥z\left(t\right){∥}_{2}}$$

where $z\left(t\right)$ represents the real signal and $\tilde{z}\left(t\right)$ represents the approximate sparse signal obtained by the recovery algorithm.

#### 3.1.1. PSL and Main Lobe Width of the Output Results

**Figure 6:**
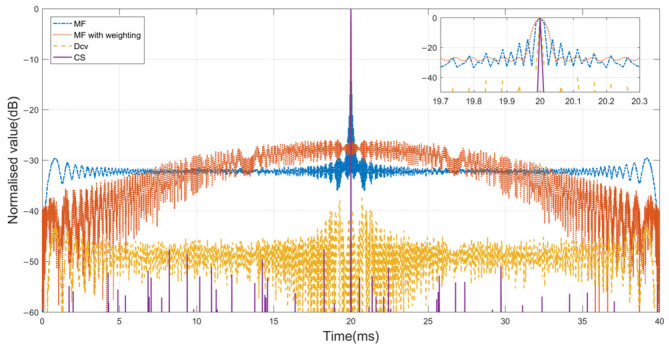
The output results with the different methods under SNR = 30 dB.

Figure 6 presents the outputs computed by the application of MF with and without the windows function, as well as the outputs derived from the application of the Dcv algorithm and the proposed method of overlapping up- and down-LFM waveforms. As shown in Table 2, a quantitative analysis was conducted by using the PSL and −3 dB main lobe width as performance metrics. A −40 dB Chebyshev window was used to effectively reduce ACF sidelobe levels, but the CCF values between the two orthogonal waveforms worsened. The deconvolution method demonstrates better sidelobe suppression at −36.82 dB, while engineering standards typically necessitate levels below −40 dB to receive a better image. Remarkably, the CS method not only achieves exceedingly lower PSL but also exhibits a narrower −3 dB main lobe width in comparison with the alternative approaches.

**Figure 7:**
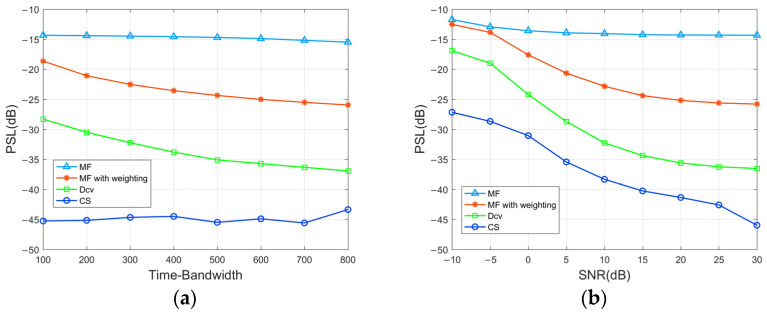
(**a**) PSLs of the output signals of the transmitted signals at different TBPs. (**b**) PSLs of the output signals at different environmental SNRs.

The investigation encompasses a comparison of the PSL results along the range dimension, considering different values of the TBP and background noisy environments. The analysis was based on averaging the results from 100 Monte Carlo simulations. Figure 7a presents a comparative depiction of the peak sidelobe levels achieved by the MF, MF with weighting, Dcv, and CS methods for different TBPs. Evidently, the PSL of the proposed method is around −45 dB and surpasses the performance of the other methods while exhibiting a minimal dependence on the TBP with effective reconstruction of target echoes from overlapped multiple waveforms. Figure 7b shows that the proposed method evidently outperforms the other methods for the output signal’s PSL with the SNR range from −10 to 30 dB. Even in a low SNR environment, CS proves the ability to effectively suppress cross-correlation noise between signals, resulting in PSL levels below −27 dB. It is worth noting that the output performance at a low SNR level is greatly influenced by noise variance. CS leverages the selection of the first m rows of the measurement matrix to determine the number of observed samples that impact the PSL when the SNR is held constant.

**Figure 8:**
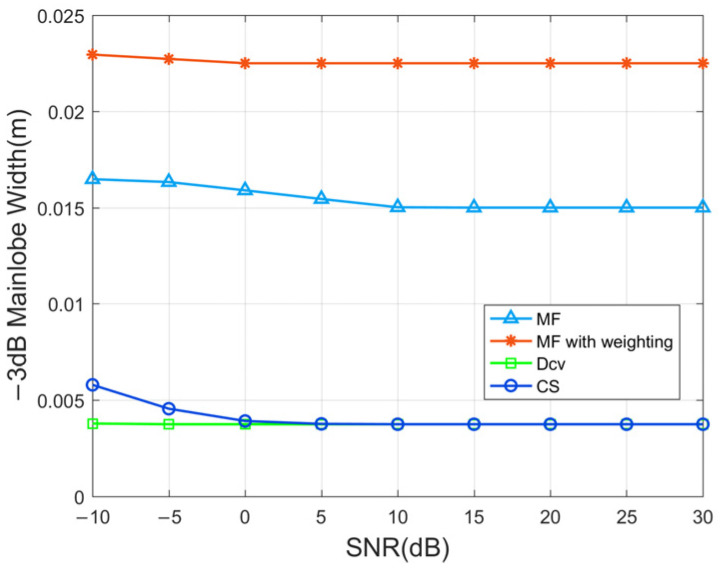
The −3 dB main lobe widths of the output signals at different SNRs.

Figure 8 illustrates the relationship between the −3 dB main lobe width of the output signals in the range dimension and the SNR levels ranging from −10 to 30 dB. As the SNR increases, there is a slight reduction in the main lobe. The adoption of wideband transmitted signals ensures a high precision level within 0.001 m. In the case of the MF technique, the main lobe typically ranges from approximately 0.015 m to 0.016 m. Although window functions effectively mitigate sidelobe levels, they contribute to a wider main lobe, reaching approximately 0.023 m. Both the CS and Dcv methods are classified as iterative optimization techniques, and the output signals can facilitate an improved determination of target positions. Moreover, the main lobe widths achieved by the CS method are approximately one-tenth of those calculated by MF.

#### 3.1.2. Imaging Results

**Figure 9:**
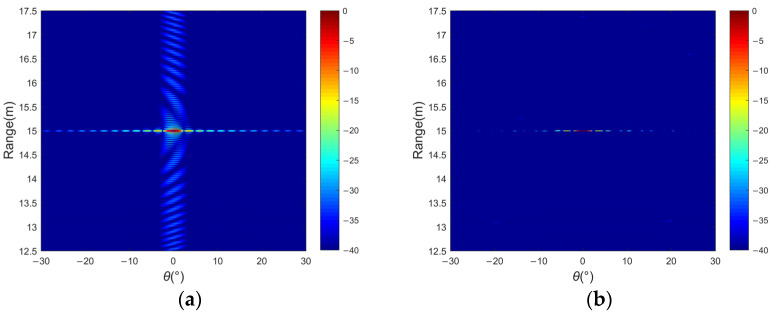
Single target of a 2D image: (**a**) MF and (**b**) CS.

Figure 9 illustrates the imaging and range profiles for single-target scenarios using both the MF and CS methods. MF exhibits relatively high sidelobe levels in the range dimension. In contrast, CS continually optimizes the sparse signal under sub-Nyquist sampling conditions, leading to significantly lower sidelobe levels and superior performance. Even in scenarios where the targets in close proximity cause interference, the proposed method accurately determines the location of the target.

**Figure 10:**
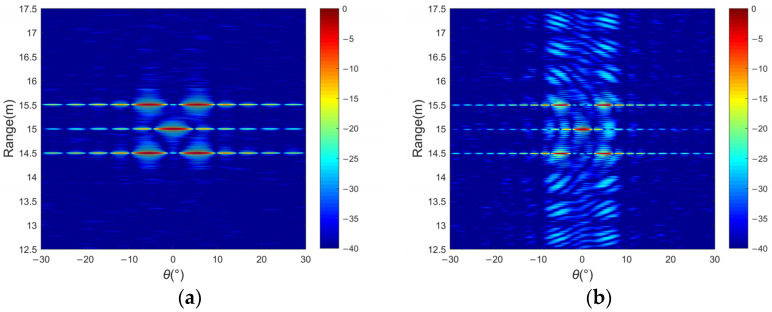

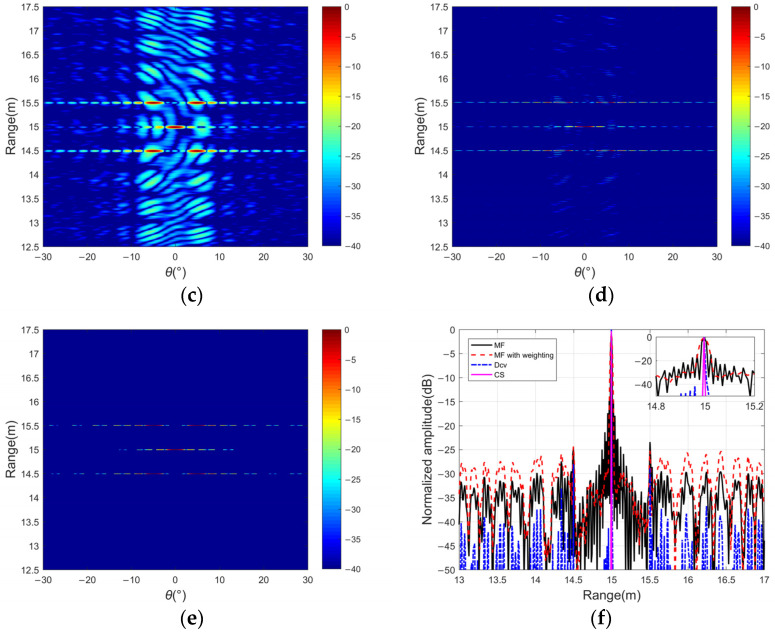
Different methods for multiple targets of a 2D sonar image and the range dimension curve: (**a**) SIMO with MF, (**b**) MIMO with MF, (**c**) MIMO with weighted MF, (**d**) MIMO with Dcv, (**e**) MIMO with CS, and (**f**) a comparison of the results in range dimension.

Figure 10 presents the two-dimensional sonar images of multiple targets. When using MF for processing overlapping echoes, sidelobe levels severely degrade target detection performance. Figure 10a shows the sonar image by transmitting a single waveform, while Figure 10b shows the image received by transmitting up and down chirp signals. Although the window function reduces the autocorrelation sidelobe value, it deteriorates the cross-correlation level, as shown in Figure 10c. Figure 10d shows that a large range of sidelobe levels of about −30 dB can be obtained in the distance dimension by the Dcv method, and the width of the main lobe narrows. Figure 10e shows the image results obtained by using the CS method. The cross-correlation noise between different waveforms is well suppressed when only using about 12% of the samples, which has certain advantages in reducing the sidelobe levels in the distance dimension of the multi-beam image. In Figure 10f, the position of the target at 15 m is clearly visible compared with the other methods. The range sidelobe level can reach −40 dB or lower, and there is no interference from other sidelobe levels; thus, it is conducive to the detection probability of the target. In addition, the utilization of a virtual aperture leads to an increased azimuthal resolution in sonar imaging compared with the scenario involving a single transmitter, as depicted in Figure 10a. By adopting large aperture arrays, better-quality sonar images can be acquired.

#### 3.1.3. The Effect of the Measurement Samples

**Figure 11:**
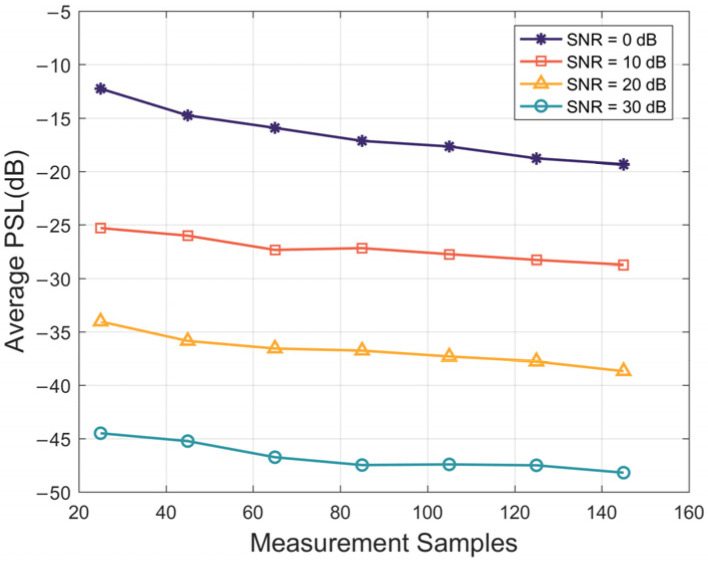
The PSL of the outputs varies with the number of observed samples under different SNRs.

The quantity of observed samples directly influences the signal reconstruction process of the algorithm. Therefore, a comprehensive analysis was conducted using 100 Monte Carlo experiments to examine the impact of different SNRs on factors such as the PSL, reconstruction error, and algorithm execution time. In Figure 11, the average PSL exhibits a consistent decreasing trend with increasing SNR. A larger number of measurement samples for the utilized DFT matrix facilitates improved signal reconstruction. At a low SNR, a higher quantity of observed samples is required to realize accurate signal recovery without disturbance of correlated noise.

**Figure 12:**
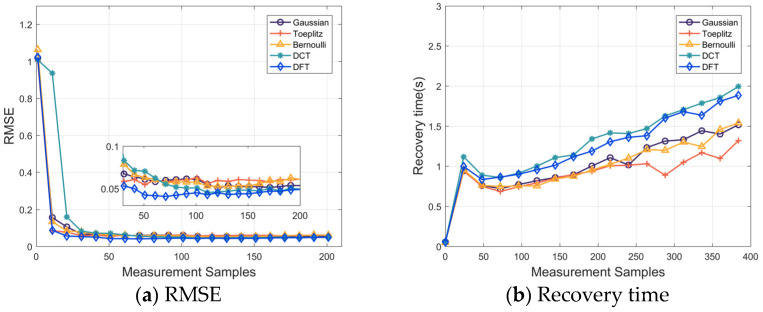
(**a**) RMSE of different observation matrices under different observation sample quantities. (**b**) The recovery time of different observation matrices under different observation sample quantities.

In Figure 12a, the observed trend indicates decreasing error with an increasing number of observed samples. After orthonormalization deterministic subsampling eliminates partial stochasticity, leading to substantial reductions in reconstruction errors compared to other matrices, the deterministic DFT matrix certifies a remarkable ability to accurately recover signals with a smaller number of samples. Figure 12b provides the runtime for signal recovery using different measurement matrices under varying numbers of observed samples. As expected, the time required for signal recovery increases with an increasing number of observed samples. In the realm of full-deterministic matrices, the DFT matrix outperforms the DCT matrix in terms of recovery time. The differences are typically on the order of seconds. It gives relative efficiency in both signal recovery accuracy and time performance.

### 3.2. Experimental Data Processing

**Figure 13:**
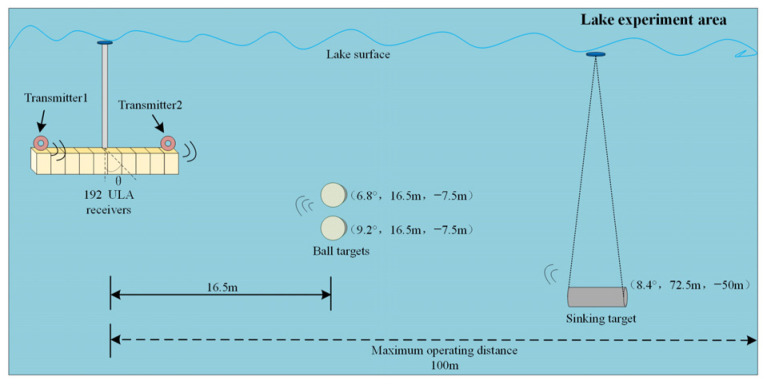
Diagram of the lake experiment scene.

To assess the effectiveness of the proposed method in practical application, a field experiment was conducted in a lake with an approximate depth of 50 m as shown in Figure 13. The array arranged for the experiment consisted of two transmitters and 192 receiver hydrophones in ULAs, with the transmitters parallel to the receivers and positioned at the ends of the receiver ULAs. The sonar wet-end was vertically fixed underwater at a depth of approximately 3 m using a long pole. Two steel spherical targets were suspended underwater at a depth of 7.5 m using cables, and the bottom target was a hollow steel cylinder. The measured distance from the sonar array to the targets was approximately 16.5 m, accounting for potential measurement errors. LFM pulses were applied to the two sides of transmitters, and the 2D imaging results processed by different methods were compared in the case of single targets and multi-targets.

#### 3.2.1. Imaging Results of Different Methods

**Figure 14:**
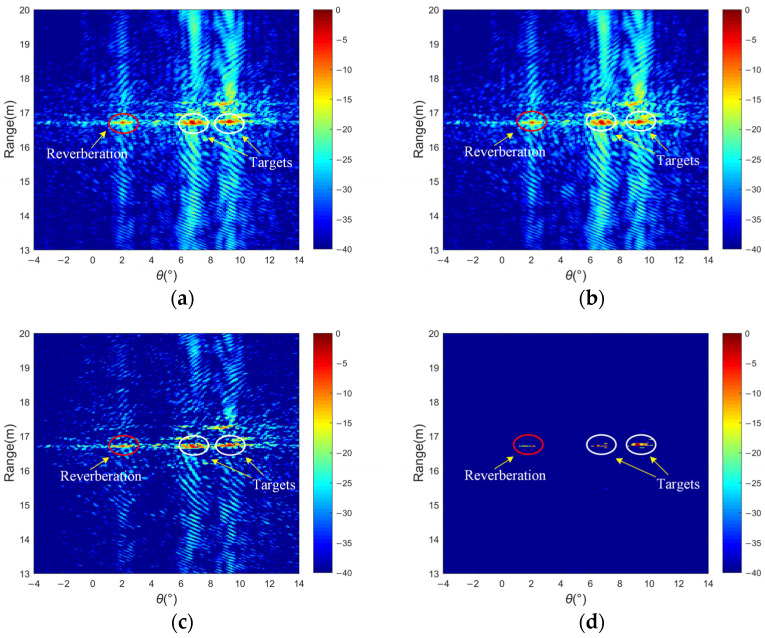
Lake experiment results of ball targets: (**a**) matched filtering, (**b**) matched filtering with weighting, (**c**) the deconvolution method, and (**d**) the proposed method.

The outcome of the lake experiment is depicted in Figure 14. The presence of clutters is observed in certain specific regions, predominantly arising from the leakage of energy in the sidelobes of the scattering regions. The two targets are situated at angles of approximately 6.8° and 9.2°, respectively, while the scatter located at −2° is attributed to the phenomenon of underwater reverberation. By using MF and weighted MF, as shown in Figure 14a,b, the resolution of the targets in terms of distance dimensions is limited due to the presence of higher sidelobe levels resulting from imperfect orthogonality among the received signals. Consequently, the sonar image does not exhibit perfect fidelity. Figure 14c displays the application of the Dcv method to process the echo signals in the range dimension. Nevertheless, some interference in the form of false peak values persists and thus adversely affects the estimation of target positions. In Figure 14d, the proposed method handles all sidelobes as false targets for iterative optimization, and the image resolution is greatly improved by iterative optimization of multi-beam data with a limited number of observed samples.

**Figure 15:**
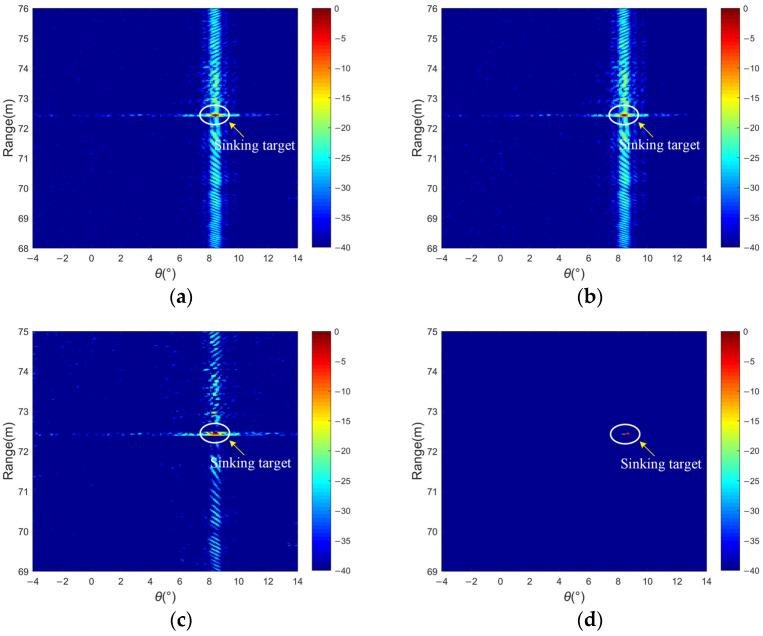
Lake experiment results of a sinking target: (**a**) matched filtering, (**b**) matched filtering with weighting, (**c**) the deconvolution method, and (**d**) the proposed method.

The results of 2D acoustic images of the bottom target in the lake experiment are displayed in Figure 15, where the target points are marked with solid white circles. Floaters marked on the lake surface ensure the location of bottom targets, and some uneven flat rocks can cause bottom disturbance. After weighting in Figure 15b, the cross-correlation sidelobe with aliasing the level of sidelobe near the main lobe is not effectively suppressed. Compared with Figure 15c,d, the proposed method effectively reduces the sidelobe interference better than Dcv, which has a larger dynamic range of −40 dB, and simultaneously realizes the high resolution of multi-beam image distance. The clutters can also be eliminated in some local regions of the target scene as noise to improve the judgment of the target location within the area of interest.

#### 3.2.2. Performance Analysis

**Figure 16:**
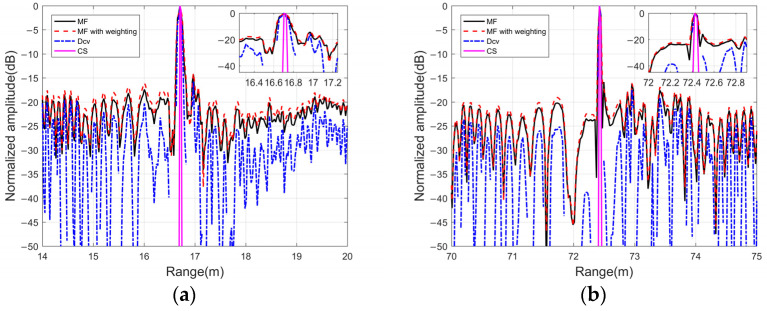
Comparison of the results of different methods in range and angle dimensions: (**a**) ball targets and (**b**) a sinking target.

Figure 16a,b presents the projection results in the distance dimensions for the target. CS shows a distinct advantage by providing a clear visualization of the peak positions for the targets, devoid of any sidelobe interference. In contrast, the traditional MF approach struggles to differentiate between closely spaced targets at similar distances. The persistence of sidelobe levels poses a challenge, as weaker targets may be obscured by the sidelobes. More importantly, it is crucial to select the number of iterations in the deconvolution process to avoid amplifying sidelobe levels due to approximation mismatch. Typically, using 10 or 20 iterations is recommended when implementing the R-L algorithm. In contrast, CS optimizes the target signal iteratively considering the noise level, and increasing the number of iterations does not lead to a deterioration in the sidelobe levels.

Furthermore, a comparison was conducted regarding the PSL and −3 dB main lobe width in the distance dimension among the different methods. Table 3 shows the PSL and −3 dB main lobe width of two kinds of targets. Compared with outputs produced by MF and Dcv, the proposed method results in much lower sidelobe levels, enabling a clearer identification of the targets. Additionally, the computed −3 dB main lobe width of the proposed method is approximately 0.02~0.03 m, which is lower than the other methods. The current study assumes a far-field model rather than practical scenarios involving near-field environments. In such cases, conventional beamforming encounters challenges due to sidelobe leakage throughout the scanning angle, including the presence of clutter in the range dimension. The proposed method handles all these sidelobes as false targets for iterative optimization, and consequently, it offers a lower PSL in terms of both target localization and sidelobe suppression. The beam sidelobe levels of the two kinds of targets have a dynamic range over −40 dB, but the interference around the two closer spherical targets is larger because of reverberation or clutters. Future investigations will also consider additional factors such as array errors and directional uncertainties in transducers to further validate the algorithm’s stability and robustness.

## 4. Conclusions

In this paper, we proposed a sparse optimization method based on CS theory to address the issue of PSL in MIMO sonar imaging systems. CS approximates LFM waveform autocorrelation outputs as sparse representations within the time domain. By selecting efficient measurement matrices and performing computations, the received signals are accurately recovered through the use of an optimization algorithm. In this process, under-sampling is realized by deterministically choosing a specific number of observed samples. The results obtained from numerical simulations and lake experiments collectively demonstrate the efficacy of the proposed method. By suppressing the cross-correlation noise among received signals, the proposed method dramatically improves the whole quality of sonar images. This improvement holds great promise for subsequent tasks such as target detection and tracking. Future research endeavors should account for more intricate environmental conditions and the algorithm’s applicability to diverse target types, including extended targets, continuous targets, and moving targets. Doppler information may be considered as auxiliary information. In the meantime, our method is currently only for LFM signals, and the processing results of different orthogonal transmitting waveforms need to be further verified in the utilization of the algorithm.

## Figures and Tables

**Table 1:** Parameter settings.

Parameters	Value
Quantity of transmitters and receivers	2 and 24
Center frequency	400 kHz
Pulse bandwidth and length	40 kHz, 20 ms
Single target	(0°, 15 m)
Multiple targets	(−5°, 14.5 m), (−5°, 15.5 m), (0°, 15 m), (5°, 14.5 m), (5°, 15.5 m)

**Table 2:** PSL and −3 dB main lobe width of the output results.

Methods	PSL (dB)	−3 dB Main Lobe Width (s)
MF	−14.34	2 × 10^−5^
MF with weighting	−25.90	3 × 10^−5^
Dcv	−36.82	2.2 × 10^−6^
CS	−47.68	1.1 × 10^−6^

**Table 3:** PSL and −3 dB main lobe width of the two kinds of targets.

Targets	Methods	PSL (dB)	−3 dB Main Lobe Width (m)
ball targets	MF	−13.85 dB	0.09
	MF with weighting	−14.03 dB	0.1
	Dcv	−15.81 dB	0.05
	CS	−59.98 dB	0.03
sinking target	MF	−15.89 dB	0.035
	MF with weighting	−16.08 dB	0.04
	Dcv	−17.39 dB	0.03
	CS	−60.01 dB	0.02

## Data Availability

Dataset available on request from the authors.
